# A comparison of basal and activity-dependent exon splicing in cortical-patterned neurons of human and mouse origin

**DOI:** 10.3389/fnmol.2024.1392408

**Published:** 2024-08-29

**Authors:** Owen Dando, Jamie McQueen, Karen Burr, Peter C. Kind, Siddharthan Chandran, Giles E. Hardingham, Jing Qiu

**Affiliations:** ^1^Edinburgh Medical School, UK Dementia Research Institute at the University of Edinburgh, Edinburgh, United Kingdom; ^2^Simons Initiative for the Developing Brain, University of Edinburgh, Edinburgh, United Kingdom; ^3^Centre for Discovery Brain Sciences, Hugh Robson Building, University of Edinburgh, Edinburgh, United Kingdom; ^4^Centre for Clinical Brain Sciences, Edinburgh Medical School, Edinburgh, United Kingdom

**Keywords:** RNA-seq-RNA sequencing, gene expression, neuronal activity, calcium signaling, evolutionary conservation and divergence, alternative splicing

## Abstract

Rodent studies have shown that alternative splicing in neurons plays important roles in development and maturity, and is regulatable by signals such as electrical activity. However, rodent-human similarities are less well explored. We compared basal and activity-dependent exon splicing in cortical-patterned human ESC-derived neurons with that in cortical mouse ESC-derived neurons, primary mouse cortical neurons at two developmental stages, and mouse hippocampal neurons, focussing on conserved orthologous exons. Both basal exon inclusion levels and activity-dependent changes in splicing showed human-mouse correlation. Conserved activity regulated exons are enriched in RBFOX, SAM68, NOVA and PTBP targets, and centered on cytoskeletal organization, mRNA processing, and synaptic signaling genes. However, human-mouse correlations were weaker than inter-mouse comparisons of neurons from different brain regions, developmental stages and origin (ESC vs. primary), suggestive of some inter-species divergence. The set of genes where activity-dependent splicing was observed only in human neurons were dominated by those involved in lipid biosynthesis, signaling and trafficking. Study of human exon splicing in mouse Tc1 neurons carrying human chromosome-21 showed that neuronal basal exon inclusion was influenced by cis-acting sequences, although may not be sufficient to confer activity-responsiveness in an allospecific environment. Overall, these comparisons suggest that neuronal alternative splicing should be confirmed in a human-relevant system even when exon structure is evolutionarily conserved.

## Introduction

Humans and mice diverged from their common ancestor approximately 80 million years ago. Nevertheless, over 90% of human genes have 1:1 orthologs in mice (Monaco et al., 2015). However, whether the expression of these orthologs in response to signaling pathways is conserved or otherwise was not well understood. A classic example of this type of response is found in CNS neurons, where electrical activity dynamically controls gene expression to influence neuronal development, neuroprotection, neurophysiological properties, and ultimately cognitive function (Soriano and Hardingham, 2007; Bell and Hardingham, 2011; Hardingham and Lipton, 2011; West and Greenberg, 2011; Hardingham et al., 2018; Lee and Fields, 2021). Neuronal activity regulates the transcription of hundreds of genes whose promoters recruit transcription factors and coactivators that are controlled by Ca^2++^-activated signaling pathways, including CREB, SRF, AP-1, FOXO, ATF4, Notch, Jacob, PGC1-α and CBP (Sheng et al., 1990; Hardingham et al., 1997; Hardingham and Bading, 1998; Cruzalegui et al., 1999; McKenzie et al., 2005; Al-Mubarak et al., 2009; Puddifoot et al., 2012; Karpova et al., 2013; Lewerenz et al., 2014).

We previously reported that activity-responsiveness of expression levels of 1:1 orthologs in mouse and human cortical neurons showed evidence of divergence (Qiu et al., 2016). The human system employed was glutamatergic cortical-patterned neurons from human embryonic stem cells (hESC^CORT^-neurons), and comparisons were made to mouse primary cortical neurons (Mus-PRIM^CORT^ neurons) at day in vitro (DIV) 4 and DIV10, as well as cortical-patterned neurons from mouse embryonic stem cells (Mus-ESC^CORT^-neurons). The rationale for employing multiple mouse neuronal preparations was to get a clearer idea of the extent of non-species-dependent differences such as developmental stage or cellular origin (primary tissue vs. stem cell). Mechanistically, we concluded that human-mouse differences in activity-responsiveness involved changes in cis-acting gene promoter regions that contain binding sites for activity-responsive transcription factors (Qiu et al., 2016). Other studies published shortly after also provided evidence of divergence of gene activity-responsiveness and showed that it could influence lineage-specific aspects of neuronal development (Ataman et al., 2016; Pruunsild et al., 2017), reviewed in (Hardingham et al., 2018).

While the basic property of whether a gene’s transcription is up- or down-regulated in neurons in response to electrical activity is of importance in determining the physiological outcome, so is post-transcriptional regulation. Eukaryotic genes possess coding exons interspersed with non-coding introns, the former of which are spliced to create protein-coding open reading frames. Many genes exhibit variable usage of exons (a form of alternative splicing) which enable a variety of related proteins to be encoded at a single genomic locus. In neurons, alternative splicing plays a key role in development (Saito et al., 2016, 2019; Wamsley et al., 2018). Moreover, many exons are subject to signal-dependent inclusion (or exclusion). Neuronal electrical activity is known to control exon usage, mediated by several RNA binding factors including the RBFOX proteins (1–3), SAM68 (Furlanis and Scheiffele, 2018; Jacko et al., 2018; Farini et al., 2020) and NOVA (Eom et al., 2013; Ibrahim et al., 2023). Moreover, activity-dependent alternative splicing regulates neurophysiological and other properties by determining the function of specific proteins (Furlanis and Scheiffele, 2018). Aberrant alternative splicing in neurons is thought to contribute to the pathogenesis of human brain disorders including Parkinson’s and Alzheimer’s diseases, and ALS-FTD (Lopez Soto et al., 2019; Li et al., 2021), and misregulation of activity-dependent exon usage is implicated in autism spectrum disorder phenotypes (Parikshak et al., 2016; Quesnel-Vallières et al., 2016; Quesnel-Vallieres et al., 2019).

However, despite mice being widely used to model human brain disease, comparisons of alternative splicing in mouse vs. human neurons are lacking. Alternative splicing has previously been compared across several species (including human and mouse) in a variety of organs, including the brain (Barbosa-Morais et al., 2012; Merkin et al., 2012; Verta and Jacobs, 2022). However, this does not give complete insight into neurons specifically since the brain is a mixture of cell types (neurons, macroglia, immune cells and vascular cells) which will have distinct alternative splicing profiles and which may contribute different proportions to the brain in different species. Furthermore, in these studies dynamic changes in alternative splicing as a result of neuronal activity was not addressed.

Here we have compared alternative splicing in cortical-patterned neurons of human and mouse origin, considering both basal levels of exon inclusion as well as changes that occur in response to neuronal activity, and focusing on exons which are directly orthologous in the human and mouse genomes. We studied splicing in human hESC^CORT^-neurons, with comparisons made to mouse primary cortical neurons (Mus-PRIM^CORT^ neurons) at day in vitro (DIV) 4 and DIV10, cortical-patterned neurons from mouse embryonic stem cells (Mus-ESC^CORT^-neurons), and also mouse primary neurons from a different brain region (hippocampus, Mus-PRIM^HIPP^ neurons). Additionally we took advantage of a mouse model Tc1, which carries a copy of human chromosome 21 (Hsa21), albeit with certain regions disrupted (O’Doherty et al., 2005; Gribble et al., 2013), enabling the study of splicing of certain human genes in a mouse neuronal environment, which can point to the importance of cis-acting DNA sequences in dictating exon splicing behavior.

## Results

### Human-mouse comparison of basal exon inclusion in cortical neurons

**FIGURE 1:**
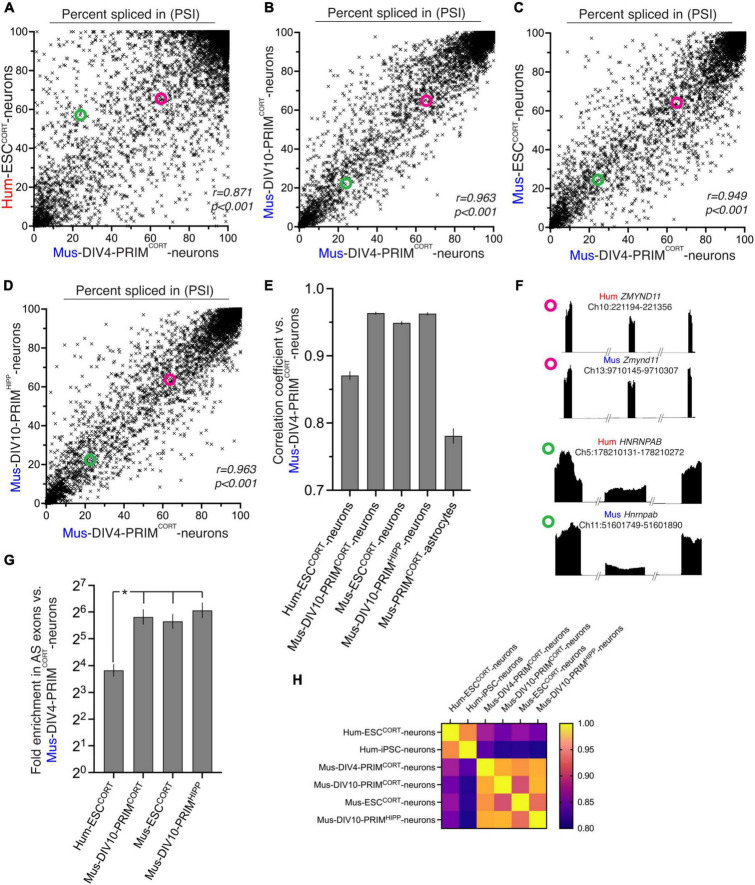
Comparison of basal exon inclusion in cortical-patterned neurons of human and mouse origin. **(A–D)** The exon inclusion ratio, otherwise known as “percent spliced in” (PSI) in DIV4 Mus-PRIM^CORT^ neurons plotted against the corresponding PSI in the indicated cell types (mean PSI, *n* = 3 independent biological replicates here and throughout the figure). All exons plotted have a 1:1 human-mouse ortholog, the mean of 3 independent biological replicates is shown. **(E)** Pearson r correlation coefficients for the comparisons made in **(A–D)**, and Supplementary Figure 2C. Error bars indicate the 95% confidence limits and in all cases *p* < 0.0001. For data relating to this figure see Source_Data.xlsx. **(F)** Examples of two 1:1 orthologous exons (coordinates relate to this exon), plus flanking exons, showing relative RNA-seq read density. One exon (from *ZMYND11*) has a similar PSI in human and mouse neurons, while one (from *HNRNPAB*) has a different PSI in human and mouse neurons. The PSI of the *ZMYND11* exon and *HNRNPAB* exon is highlighted in the scatter graphs **A–D** with magenta and green circles, respectively. **(G)** Fold enrichment of exons classed as alternatively spliced in DIV4 Mus-PRIM^CORT^ neurons in exons which are also classed as alternatively spliced in the indicated neuronal cell types (defined as 80 > mean PSI > 20, *n* = 3). Error bars indicate the 95% confidence limits of the enrichment factor. In all cases *p* < 0.0001 (Fisher’s exact test). *P < 0.05 (normal approximation to difference in log odds ratios). **(H)** A heat map summary showing the correlation coefficients of all possible pairwise comparisons as indicated.

We compared basal exon inclusion in human vs. mouse cortical neurons, before analyzing any activity-dependent changes. We first made a genome-wide comparison between basal exon inclusion levels between Hum-ESC^CORT^ and DIV4 Mus-PRIM^CORT^ neurons that we had previously performed RNA-seq on Qiu et al. (2016). Comparisons were restricted to “orthologous” exon inclusion/exclusion events, namely those whose upstream exon end, downstream exon start, and start and end of the alternatively spliced exon could be matched to within ten base-pairs, after translating co-ordinates between the mm10 and hg38 genome assemblies (28.9% and 27.4%, respectively, of mouse and human events detected in 1:1 orthologous genes). We used the “percent spliced in” (PSI) term to describe exon inclusion levels: 100% is a constitutively spliced exon; 0% is a constitutively skipped exon. We observed a significant correlation between Hum-ESC^CORT^ and DIV4 Mus-PRIM^CORT^ neurons when comparing the PSI of orthologous exons (Figures 1A, E). For each sample set we also classified every exon as primarily included (PI, PSI > 80), primarily skipped (PS, PS < 20) or alternatively-spliced (AS, 80 > PSI > 20). In Hum-ESC^CORT^ neurons, alternatively-spliced exons were enriched 13-fold in those exons also alternatively-spliced in DIV4 Mus-PRIM^CORT^ neurons (Figure 1G). Collectively this suggests that the basal level of orthologous exon inclusion in human and mouse cortical neuronal mRNA transcripts exhibits significant conservation, although the correlation was far from perfect. Figure 1F illustrates two orthologous exons (from *ZMYND11* and *HNRNPAB*), one of which (from *ZYMYND11*) has a similar PSI in Hum-ESC^CORT^ and DIV4 Mus-PRIM^CORT^ neurons, and one (from *HNRNPAB*) is quite different.

We wanted to gain a better indication as to whether the imperfect correlation in exon inclusion levels observed (Figure 1A) may be in part due to evolutionary divergence. Non species-specific differences could in theory be responsible, such as the two populations of neurons being at a different developmental stage, derived from different sources (embryonic stem cell line vs. primary tissue), or even simple experimental variation. We therefore performed identical analyses between DIV4 Mus-PRIM^CORT^ neurons and more mature DIV10 Mus-PRIM^CORT^ neurons as well as with mouse ES cell-derived cortical neurons (Mus-ESC^CORT^-neurons) to determine the approximate influence of developmental stage (DIV4 vs DIV10) and tissue origin (primary vs. stem cell) on PSI. We had previously subjected these samples to RNA-seq analysis (Qiu et al., 2016). These inter-mouse comparisons showed a higher correlation between each other (Figures 1B, C, E) and higher enrichment of alternatively spliced genes than that observed in the Hum-ESC^CORT^ vs. DIV4 Mus-PRIM^CORT^ comparison (around 50-fold, Figure 1G). We also compared the PSI in our DIV4 Mus-PRIM^CORT^ neurons to that in DIV10 neurons generated and analysed by a different laboratory and from a different brain region [hippocampus, DIV10 Mus-PRIMHIPP, (Quesnel-Vallières et al., 2016)]. We observed a good correlation and strong enrichment: similar to that observed between mouse neuronal preparations made in our laboratory (Figures 1D, E, G).

Aware that our comparisons thus far involve only one human-derived dataset, we compared basal exon PSI levels in our samples to those calculated from a published transcriptome (RNA-seq) of human iPSC-derived neurons (Pruunsild et al., 2017). We found that exon PSI levels in these human iPSC-derived neurons correlated well with our Hum-ESC^CORT^ neurons that was substantially stronger than comparing to our mouse neuronal samples (Supplementary Figure 2 and Figures 1H). Thus, two independently derived human neuron samples show stronger correlation with each other than with any of the mouse samples.

A heat map summary showing the correlation coefficients of all possible pairwise comparisons of the data relating to the six aforementioned neuronal samples (four mouse, two human) illustrates that all intra-mouse and intra-human correlations are higher than all human-mouse correlations (Figure 1H). This suggests that maturation state and origin (tissue vs. stem cell) or the particular cell line chosen are unlikely to account for all of the changes in splicing observed between Hum-ESC^CORT^ and mouse neurons.

We next wanted to determine whether the inter-species differences in exon PSI were diminished when only considering genes whose expression is similar. Taking only data from genes expressed at similar levels in Hum-ESC^CORT^ and DIV 4 mouse neurons (within 20% in either direction) we observed similar correlation coefficients (Supplementary Figure 2A) as the full data set (Figure 1E), with mouse-mouse comparisons stronger than human-mouse. This suggests that divergence in exon PSI is not associated with divergence in expression level.

Although our study is focussed on exon inclusion events, which represent the majority of AS events in neurons (> 70%), we wanted proof-of-principle that other types of event follow a similar pattern of divergence/conservation, choosing “retained intron” events, which represent around 5% of AS events. Despite there being far fewer events, there were sufficient to show a correlation in “percent retained intron” between DIV4 mouse neurons and other mouse neuronal populations, and a weaker correlation between DIV4 mouse neurons and Hum-ESC^CORT^ neurons (Supplementary Figures 1A–E).

Collectively these data support a model whereby basal PSI of orthologous exons in mRNAs from human vs. mouse cortical neurons exhibit some evolutionary divergence. Interestingly, the mouse cortical neuronal exon usage pattern was found to be more similar to that in human cortical neurons than that in mouse cortical astrocytes (Figures 1E, F and Supplementary Figure 2C). That the splicing landscape in mouse neurons is more similar to human neurons than mouse astrocytes (a closely related neural cell) illustrates that there is significant conservation in cortical neuronal alternative splicing as well as the aforementioned divergence. It also underlines the usefulness in studying alternative splicing in individual cell types rather than whole tissues such as brain (Barbosa-Morais et al., 2012; Merkin et al., 2012; Verta and Jacobs, 2022).

### Human-mouse comparison of activity-dependent alternative splicing

**FIGURE 2:**
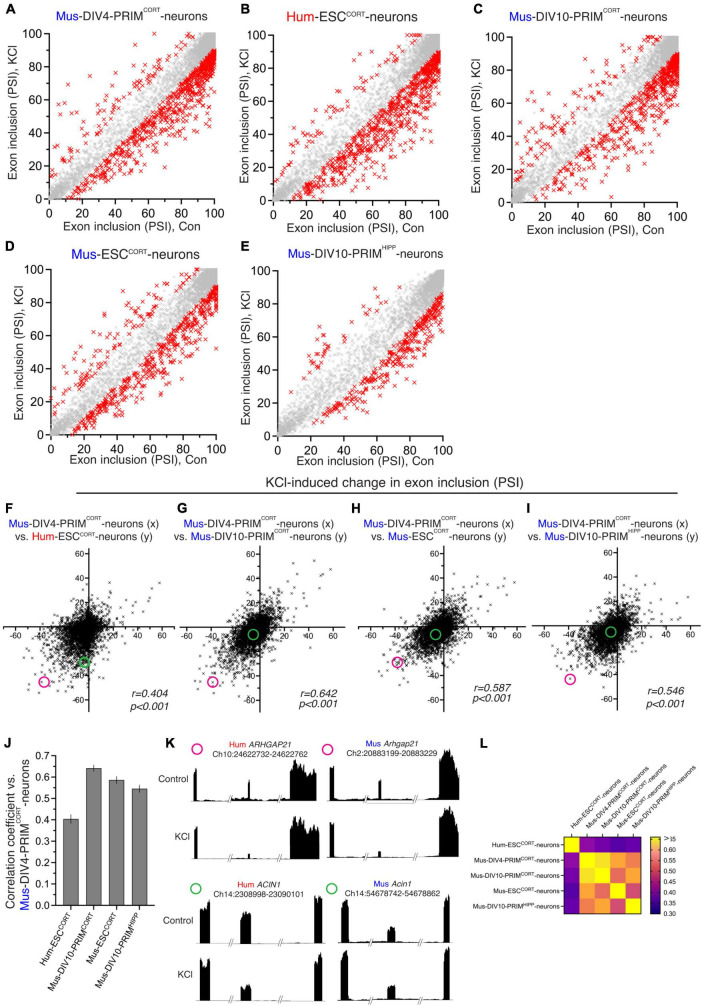
Comparison of activity-dependent alternative splicing in human and mouse cortical neurons. **(A–E)** For the indicated neuronal preparations, PSI of exons in control neurons is plotted against that in KCl-stimulated neurons [**(A–D)**: 4h; E: 3h-data were generated by another lab (Quesnel-Vallières et al., 2016)]. All exons plotted have a 1:1 human-mouse ortholog. Red crosses indicate a significant difference in PSI (p < 0.05, read count for exon inclusion or exclusion in all samples > 5, PSI difference > 10, *n* = 3). **(F)** The KCl-induced change in PSI in DIV4 Mus-PRIM^CORT^ neurons is plotted against the corresponding change in Hum-ESC^CORT^ neurons. **(G–I)** The KCl-induced change in PSI in exons in DIV4 Mus-PRIM^CORT^ neurons is plotted against the corresponding change in the indicated cell types. **(J)** Correlation coefficients for the comparisons made in **(F–I)**. Error bars indicate the 95% confidence limits and in all cases. p < 0.0001 For data points relating to this figure see Source_Data.xlsx. **(K)** Examples of two 1:1 orthologous exons (coordinates relate to this exon), plus flanking exons, showing relative RNA-seq read density. One exon (from *ARHGAP21*) has a similar KCl-induced PSI change in human and mouse neurons, while one exon (from *ACIN1*) is only subject to activity-dependent alternative splicing in human neurons. The KCl-induced PSI change of the *ARHGAP21* exon and *ACIN1* exon is highlighted in (F,G,H and I) with a magenta and a green circle, respectively. **(L)** A heat map summary showing the correlation coefficients of all possible pairwise comparisons as indicated.

We next studied changes in exon inclusion within neurons in response to to L-type Ca^2+^ channel activation, an important mediator of activity-dependent gene regulation (Sheng et al., 1990; Bito et al., 1997; Deisseroth et al., 2003; West and Greenberg, 2011; Wheeler et al., 2012; Ma et al., 2014). To do this, hESC^CORT^-neurons and DIV4 Mus-PRIM^CORT^ neurons were treated ± KCl-induced membrane depolarization in the presence of the L-type Ca^2+^ channel agonist FPL64176, plus NMDA receptor antagonist MK-801 (hereafter KCl) for 4 h (MK-801 is used as standard to prevent any excitotoxicity and associated gene expression (Schramm et al., 1990; Ramnath et al., 1992; Xia et al., 1996; Wahl et al., 2009; Zhou et al., 2009; Gupta et al., 2013). After 4 h RNA was harvested and RNA-seq performed, followed by analysis of KCl-induced changes in exon usage. In both DIV4 Mus-PRIM^CORT^ neurons (Figure 2A) and hESC^CORT^-neurons (Figure 2B) splicing levels changed significantly in 800–900 orthologous exons, with KCl treatment causing an increase in exclusion of certain exons, and inclusion of others. Others have reported previously that in mouse hippocampal neurons, activity-induced exclusion/skipping of exons in response to neuronal activity is more prevalent than activity-induced increases in exon inclusion (Quesnel-Vallières et al., 2016). This is also something we observe, not only with mouse neurons but human neurons too (Figures 2A, B). Since neuronal activity also regulates genes at the transcriptional level, we plotted changes in exon PSI against the Log2 fold change at the gene mRNA level. We found no correlation (Supplementary Figure 3) which indicates that changes in splicing are not coupled to changes in gene expression, (which is expected as they are mediated by distinct cis-acting sequences).

Similar alternative splicing analyses were then performed for DIV10 Mus-PRIM^CORT^ neurons ± KCl (Figure 2C), Mus-ESC^CORT^-neurons ± KCl (Figure 2D) and on RNA-seq data obtained by another laboratory: a 3h KCl stimulation of DIV10 Mus-PRIM^HIPP^ neurons (Quesnel-Vallières et al., 2016; Figure 2E). Plotting KCl-induced changes in PSI in hESC^CORT^ vs. DIV4 Mus-PRIM^CORT^ neurons revealed a correlation, albeit quite weak (Figure 2F). Figure 2K shows examples of RNA-seq read density in two 1:1 orthologous exons for both species ± KCl treatment. One exon (from *ARHGAP21*) has a similar KCl-induced PSI change in human and mouse neurons, while one exon (from *ACIN1*) is only subject to activity-dependent alternative splicing in human neurons. Globally, the correlation between KCl-induced changes in PSI of exons in DIV4 Mus-PRIM^CORT^ neurons vs. DIV10 Mus-PRIM^CORT^ neurons and vs. mESC^CORT^-neurons are significantly higher (Figures 2G, H, J). We also compared KCl-induced changes in PSI in DIV10 Mus-PRIM^CORT^ neurons with activity-dependent changes in PSI calculated from similar RNA-seq data obtained by another laboratory: a 3h KCl stimulation of DIV10 mouse hippocampal neurons (Quesnel-Vallières et al., 2016), also revealing a correlation similar to our comparisons of DIV10 Mus-PRIM^CORT^ neurons vs. DIV4 Mus-PRIM^CORT^ neurons and mESC^CORT^-neurons (Figures 2I, J). A heat map showing the correlation coefficients of all possible pairwise comparisons of the data relating to the five neuronal samples (four mouse, one human, Figure 2L) illustrates that all inter-mouse correlations are higher than all human-mouse correlations (Figure 1H), indicative of species-specific differences in activity-dependent splicing in human vs. mouse cortical neurons.

**FIGURE 3:**
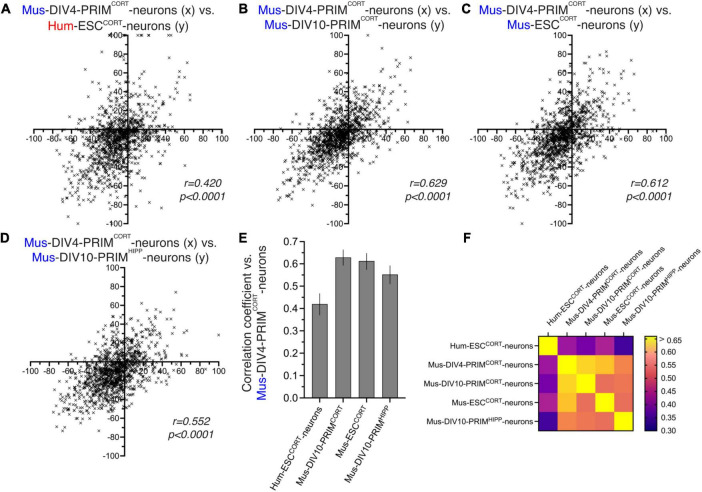
Comparison of activity-dependent alternative splicing as a percentage of possible change. **(A–D)** For exons classed as alternatively spliced (80 > PSI > 20) in DIV4 Mus-PRIM^CORT^ neurons, the effect of KCl stimulation on PSI was calculated as a percentage of the maximum possible PSI change and plotted (x-axis) against the corresponding value for the other cell types (y-axis) as indicated. **(E)** Correlation coefficients for the comparisons made in **(A–D)**. Error bars indicate the 95% confidence limits and in all cases *p* < 0.0001. For data points relating to this figure see Source_Data.xlsx. **(F)** A heat map summary showing the correlation coefficients of all possible pairwise comparisons as indicated.

One factor to consider when assessing differences in KCl-induced changes in PSI in different neuronal preparations is that the magnitude of change may be influenced by differences in basal PSI, which we know shows human-mouse differences (Figure 1). For example, a mouse exon with a basal PSI of 40 that increases upon KCl stimulation has a theoretical maximal PSI change of 60, whereas if the orthologous human exon has a basal PSI of 70 then the maximal PSI change possible is only 30. We therefore performed additional comparisons of KCl-induced inclusion level differences calculated as a % of the maximum possible inclusion level difference. We restricted our analysis to orthologous exons where basal PSI was > 20 and < 80 to eliminate excessive skewing of the data caused by modest absolute changes in PSI giving very high percentage figures (e.g., a PSI changing from 95 to 98 would give a figure of 60%). Our comparisons (Figures 3A–F) mirrored those made in Figures 2F–J and yielded similar results: KCl-induced changes in exon inclusion in hESC^CORT^ vs. mouse neurons showed significant correlation, but it was weaker than when comparing the different mouse neuronal preparations with each other. These observations are consistent with their being evolutionary divergence in activity-dependent alternative splicing of orthologous exons between mice and humans.

### Ontology of human-specific vs. conserved activity-dependent alternatively spliced genes

There are several examples of activity-dependent changes in exon inclusion influencing the function of the protein encoded by the alternatively spliced transcript (Furlanis and Scheiffele, 2018). This can require that an exon encodes a functionally autonomous part of a protein so it can be included or excluded to alter a protein’s function without causing non-specific loss of function (e.g. through protein misfolding or removing part of a key structural domain). We reasoned that the organization of exons in a gene is more likely to be conserved in those exons subject to signal-dependent regulation. Indeed, taking exons that are subject to activity-dependent regulation in mouse neurons (DIV4 and DIV10 Mus-PRIM^CORT^, mESC^CORT^-neurons) we observed that they are enriched 2.7-fold in 1:1 human-mouse orthologs (*p* < 0.0001 (Fisher’s exact test)). The fact that exons subject to activity-dependent regulation in mouse neurons are more likely to have a direct human ortholog points to evolutionary pressure to maintain genetic structure where the exon is subject to alternative splicing.

**FIGURE 4:**
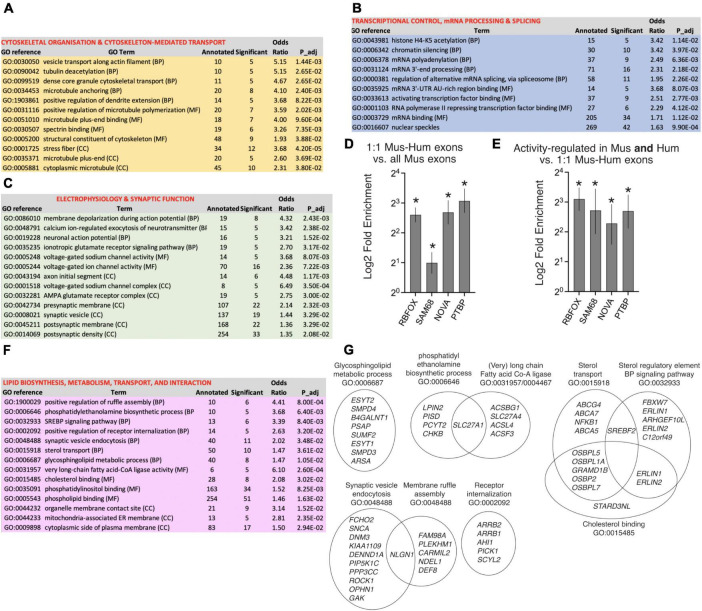
Ontology of genes subject to human-mouse conserved and “human-specific” activity-dependent exon usage. **(A–C)** Selected GO terms are shown which are enriched (Fisher’s weighted *p* < 0.05) in genes that have one or more 1:1 human-mouse orthologous exon which are subject to activity-dependent inclusion/exclusion in both Hum-ESC^CORT^ neurons and in one or more of our mouse cortical neuronal preparations (DIV4 and DIV10 Mus-PRIM^CORT^, mESC^CORT^-neurons). 782 genes contain exons that qualify as being regulated in a conserved manner by the above criteria, out of a background of 8039 genes (defined as 1:1 orthologous genes possessing ≥ 1 orthologous exons expressed in human and mouse neurons). The nature of the GO term is shown (BP, Biological Process; MF, Molecular Function; CC, Cellular Component). **(D,E)** Enrichment tests were performed for RBFOX and SAM68 target cassette exon splicing events (Jacko et al., 2018; Farini et al., 2020) (see Methods, *p < 0.0001 (Fisher’s exact test)). For **(D)** the presence of these target exons was compared between the set of all exons expressed in mouse neurons, and the set of exons expressed in mouse neurons for which there is a 1:1 human ortholog. For **(E)** the presence of these target exons was compared between the set of 1:1 orthologous exons subject to activity-dependent splicing in both human and mouse neurons [as per **(A–C)**], and the whole set of expressed 1:1 orthologous exons. **(F,G)** Selected GO terms are shown which are enriched (Fisher’s weighted *p* < 0.05) in genes that have a 1:1 human-mouse orthologue which are subject to activity-dependent alternative splicing in human neurons but not mouse neurons. In **(G)** the genes within selected GO terms that account for the enrichment are shown, and any genes in more than one GO term indicated by the overlapping nature of the Venn diagram. Note that while selected pathways are shown in this figure, all significantly enriched pathways are shown in Source_Data.xlsx.

Taking genes containing orthologous exons subject to activity-dependent changes in PSI in both human and mouse neurons, ontological analysis revealed three main functional areas (Figures 4A–C). Prominent processes and functions are associated with cytoskeletal organization and transport along cytoskeletal tracks (Figure 4A). The second major area is in the control of gene expression including transcriptional control, epigenetic regulation, and post-transcriptional mRNA processing, including polyadenylation and RNA splicing itself (Figure 4B). A third prominent area of conserved activity-responsive AS functions involve synaptic signaling and action potential firing (Figure 4C), and associated subcellular components such as synaptic vesicles, the pre- and post-synaptic membrane, and specialist structures including the post-synaptic density, AMPA receptor complex and the axon initial segment. These processes have been highlighted recently as subject to regulation by activity-dependent AS, particularly in the context of homeostatic plasticity in mice (Iijima and Yoshimura, 2019; Thalhammer et al., 2020) and our data suggests that activity-dependent AS may play a similar role in human neurons.

We were also interested in identifying putative regulators of this conserved group of activity-dependent exons. Dynamic changes in exon usage in developing neurons are controlled by several RNA binding factors, prominent among them being the RBFOX proteins (1–3), SAM68, NOVA, and PTBP (Eom et al., 2013; Saito et al., 2016, 2019; Furlanis and Scheiffele, 2018; Jacko et al., 2018; Wamsley et al., 2018; Farini et al., 2020; Ibrahim et al., 2023), with SAM68 and NOVA particularly implicated in activity-dependent exon usage (Iijima et al., 2011; Eom et al., 2013; Farini et al., 2020; Ibrahim et al., 2023).

Consistent with conserved importance of exons regulated by these factors, their targets are enriched in orthologous exons compared to non-orthologous ones (Figure 4D). Moreover, even after allowing for this enrichment, orthologous exons exhibiting activity-dependent changes in PSI in both human and mouse neurons are further enriched in targets of these splicing factors (Figure 4E). Thus, exons regulated by RBFOX, SAM68, NOVA, and PTBP are both conserved in terms of gene structure and additionally form a significant element of conserved exons subject to activity-dependent exon inclusion in human and mouse neurons.

We also wanted to determine whether genes subject to “human-specific” activity-dependent exon inclusion were enriched in any specific biological processes. We studied genes that have a 1:1 human-mouse orthologue which are subject to human neuron-specific activity-dependent alternative splicing. “Human-specific” regulation was defined as genes that in Hum-ESC^CORT^ neurons had 1 or more exons whose PSI in the mature transcript changed > 10% (up or down, p < 0.05) upon KCl treatment, but whose mouse ortholog did not meet these criteria in any of the mouse neuronal preparations used in this study. 1104 genes were found to be subject to “human-specific” activity-dependent alternative splicing (by the criteria above) and were subject to ontological analysis. Biological processes and molecular function terms enriched in genes subject to human-specific activity-dependent alternative splicing are dominated by lipid biology (Figures 4F, G). This includes gene sets involved in lipid/phospho-lipid biosynthesis, regulation of lipid biosynthesis (e.g. signaling to the sterol regulatory element) and lipid interaction, as well as lipid transport and processes associated with lipid bilayer dynamics such as synaptic vesicle endocytosis and membrane ruffle assembly. The prominence of a wide range of gene sets relating to lipid biology (Figures 4F, G) is also clear when reviewing all pathways that are significantly enriched (see Source_Data.xlsx). The functional consequences of activity-dependent AS of so many lipid pathway genes will require further investigation.

### Cis vs. trans determinants of basal and activity-dependent alternative splicing

**FIGURE 5:**
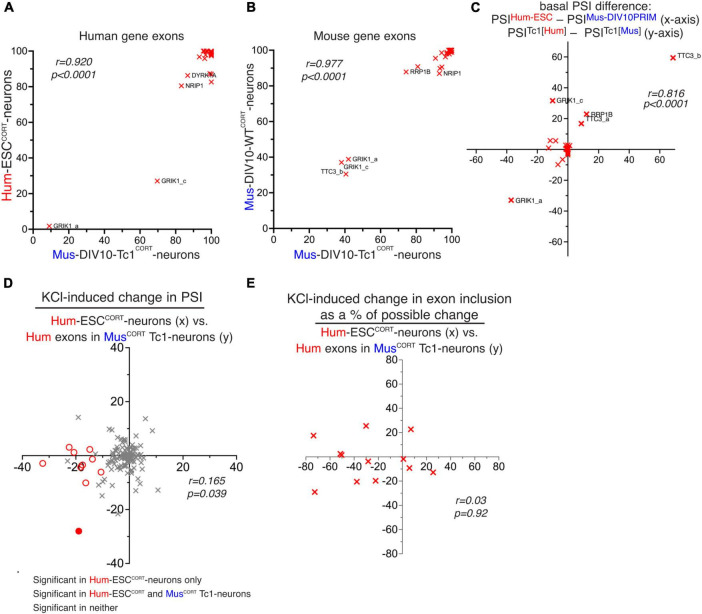
Study of human and mouse gene basal and activity-dependent alternative splicing in mouse Tc1 neurons. **(A)** PSI of hCh21 exons (with a 1:1 mouse/human ortholog) in Hum-ESC^CORT^ neurons vs. the PSI of the same human exon in mouse Tc1 neurons. **(B)** PSI of the mouse orthologs of the exons from Figure 5A in mouse Tc1 neurons (x-axis) vs. PSI of the same exons in DIV10 Mus-PRIM^CORT^ neurons. **(C)** A comparison of the difference in basal PSI in orthologous exons within mouse Tc1 neurons compared to the difference in the same exons between mouse (DIV10 Mus-PRIM^CORT^) and human (hESC^CORT^) neurons. Correlation coefficient r is shown. **(D)** A comparison of KCl-induced PSI changes in human Ch21 exons in Hum-ESC^CORT^ neurons vs. mouse Tc1 neurons. **(E)** For alternatively spliced exons (80 > PSI > 20) the effect of KCl stimulation on PSI as a percentage of the maximum possible PSI change was compared between Hum-ESC^CORT^ neurons vs. mouse Tc1 neurons. For data points relating to this figure see Source_Data.xlsx.

Our human-mouse and mouse-mouse comparisons suggest that both basal exon inclusion levels and activity-dependent changes in exon inclusion show a degree of evolutionary divergence. We wanted to get an indication as to whether putative species-specific differences are due to changes in cis-acting factors (i.e. DNA sequence) or trans-acting factors, such as splicing factors or signal transduction machinery. RNA-seq data from cortical neurons cultured from the Tc1 transchromosomic mouse strain (O’Doherty et al., 2005) was analysed, since these neurons contain an extrachromosomal copy of human chromosome 21 (O’Doherty et al., 2005), albeit an incomplete copy (Gribble et al., 2013), enabling us to assess the PSI of human chromosome 21 exons in parallel with their mouse ortholog in the same cellular environment. We used our *in silico* species-specific RNA-seq read analysis workflow (Qiu et al., 2018) to distinguish between human and mouse genome-derived RNA-seq reads within the same RNA sample. We first considered the basal PSI of the human exons in Tc1 mouse neurons and compared them to the PSI of those same exons in their normal human cellular environment (hESC^CORT^-neurons). Of the 45 hCh21 exons which both had a 1:1 mouse orthologue and passed an expression level threshold, there was a significant correlation comparing PSI of human exons in Tc1 cortical neurons vs. the human cellular environment of Hum-ESC^CORT^ neurons (Figure 5A). As expected, the corresponding mouse exons showed near-identical PSI in Tc1 cortical neurons vs. DIV10 Mus-PRIM^CORT^ neurons (Figure 5B). We also wanted to determine whether human-mouse differences in PSI of orthologous exons observed when comparing the transcriptomes of human and mouse neurons were also observed when those exons were studied in the same cellular environment (mouse Tc1 neurons). We observed a correlation between human-mouse differences in PSI when studying them in their own cellular environment vs. a common Tc1 cellular environment (Figure 5C). This supports a model whereby basal PSI is dictated by cis-acting DNA elements and that human-mouse differences may be driven at least in part by divergence in cis-acting DNA elements.

We next investigated activity-dependent changes in splicing in human Ch21 exons within the mouse Tc1 neurons, and this was compared to changes of the same exons in Hum-ESC^CORT^ neurons. This revealed a very poor correlation (Figure 5D). There were 10 hCh21 exons (spanning 9 different genes) that undergo activity-dependent changes in inclusion in Hum-ESC^CORT^ neurons (PSI change ≥ 10 in any direction, *p* < 0.05) and whose regulation could be studied in mouse Tc1 neurons. Only 1 out of those 10 human exons were controlled by KCl treatment of Tc1 neurons (Figure 5D). We also performed additional comparisons of activity-induced splicing changes calculated as a percentage of the maximum possible change, restricting our analysis to orthologous exons where basal PSI was > 20 and < 80 as before (a similar approach to that taken in Figure 3). This also revealed no correlation between activity-dependent changes in human exon inclusion in a human (Hum-ESC^CORT^) vs. mouse (Tc1) cellular environment (Figure 5E).

Thus, unlike basal inclusion levels of human exons which in mouse Tc1 neurons appeared to correlate quite well with that observed in Hum-ESC^CORT^ neurons, activity-dependent changes in human gene exon inclusion were not recapitulated in mouse Tc1 neurons. This suggests that cis-acting DNA elements may not be sufficient to direct activity-dependent alternative splicing and “trans-acting factors” such as a human neuron’s activity-responsive signaling or splicing machinery may be required. However, the relatively small number of activity-responsive exons studied means that we cannot rule out that certain activity-responsive human exons can also be similarly regulated in a mouse cellular environment. Of the activity-responsive human exons that could be analyzed in Tc1 neurons none were putative targets of RBFOX, SAM68, NOVA or PTBP. It is possible that conserved exon targets of these factors can be controlled by mouse splicing factors directed by cis-acting binding sites for these factors, although whether these splicing factors have cross-species activity is not clear.

### Concluding remarks

To conclude, our study indicates that there is significant conservation of both basal and activity-dependent exon usage between cortical-patterned human and mouse neurons. The classes of genes previously identified as being subject to activity-dependent alternative splicing in mouse neurons: synaptic, electrophysiology, cytoskeletal (Furlanis and Scheiffele, 2018; Jacko et al., 2018; Iijima and Yoshimura, 2019; Farini et al., 2020), also feature strongly in genes whose splicing is similarly regulated in human neurons. These genes whose activity-dependent splicing is conserved are also enriched in regulatory targets of splicing factors such as RBFOX and SAM68. However our study also supports the notion that there are both quantitative and qualitative differences in orthologous exon usage in human neurons, compared to their mouse ortholog. Moreover, differences in both basal exon usage and the activity-dependency of exon usage are apparent, although cis-acting sequences may be sufficient to drive the former, but not the latter. It is conceivable that the functional impact of neuronal activity on human forebrain neurons is different to those from mice, and that these differences may arise from alternative exon usage and not just differential regulation at the transcriptional level (Hardingham et al., 2018; Qiu et al., 2018). The prominence of lipid biology in genes with exons subject to human-specific control by neuronal activity is intriguing and provides a basis for further functional investigation.

## Materials and methods

### Splicing analysis

We used RNA-seq data from the following accessions: E-MTAB-5489 (Qiu et al., 2016), E-MTAB-5514 (Hasel et al., 2017), GSE89984 (Quesnel-Vallières et al., 2016) and GSE88773 (Pruunsild et al., 2017). Samples containing RNA-seq reads from only a single species were mapped to their respective genome using the STAR version 2.7.0f (Dobin et al., 2013); reads were mapped to the primary assemblies of the human (hg38) or mouse (mm10) reference genomes contained in Ensembl release 99 (Cunningham et al., 2022). Samples containing RNA-seq reads derived from both the human and mouse genomes (or single-species samples which were to be compared with these) were processed with Sargasso version 2.1 (Qiu et al., 2018) to disambiguate reads between the two species, using a conservative filtering strategy, to prioritize minimizing the number of read mis-assigned to the wrong species. In order to measure levels of exon inclusion, and differences in exon inclusion between experimental conditions, data were then processed with the differential splicing tool rMATS, version 4.1.0 (Shen et al., 2014), focusing on the “skipped exon” category of splicing events. Significance for differential inclusion events was generally defined as *p* < 0.05, read count for exon inclusion or exclusion in all samples > 5, inclusion level difference > 10 PSI (percent spliced in).

To match orthologous skipped exon events, the co-ordinates of mouse events were transformed from mm10 to hg38 co-ordinates using the command-line version of the UCSC liftOver tool.^1^ Mouse and human exon inclusion/exclusion events were then considered to be orthologous if the human co-ordinates and lifted mouse co-ordinates of the upstream exon end, downstream exon start, and the start and end of the alternatively spliced exon could be matched to within ten base-pairs.

### Enrichment analysis

Enrichment analyses were performed as follows. For gene ontology enrichments, a background gene set was constructed consisting of all human genes for which an event with average read count for exon inclusion or exclusion over all samples greater than 5 was tested for differential splicing, which had a 1:1 orthologous mouse gene, and for which the mouse gene had an event with average read count > 5 tested in at least one of the DIV4, DIV10 or mESC KCl vs basal comparisons. Then gene ontology enrichment was tested in (i) those human genes which had a significant differential splicing event (according to the definition above), for which the 1:1 orthologous mouse gene had a significant differential splicing event in at least one of the DIV4, DIV10 or mESC KCl vs basal comparisons; (ii) those human genes which had a significant differential splicing event for which the 1:1 orthologous mouse gene did not have a significant differential splicing event in any of the DIV4, DIV10 or mESC KCl vs basal comparisons. Gene ontology enrichment analyses were performed using topGO, R package version 2.42.0 (Alexa et al., 2006).

At the level of splicing events themselves, enrichments for targets of particular splicing factors were tested using Fisher’s exact test. For each splicing factor three enrichment tests were performed: (i) in the background of all mouse events with average read count for exon inclusion or exclusion over all samples greater than 5 which were tested for differential splicing in at least one of the DIV4, DIV10 or mESC KCl vs. basal comparisons, enrichment for those events with an orthologous human event with average read count greater than 5 which was tested for differential splicing; (ii) in the background corresponding to the enrichment set in (i), enrichment for those events which were significant in the human KCl vs basal comparison, and also in at least one of the mouse DIV4, DIV10 or mESC KCl vs basal comparisons. Enrichment tests were performed for (i) Rbfox target cassette exon splicing events determined by RNA-seq profiling after 10 days of maturation in Rbfox triple KO vs. WT neurons as reported (Jacko et al., 2018); (ii) the union of Sam68 cassette exon splicing events determined by RNA-seq from Sam68 KO vs. WT neurons at P1 and P10 (Farini et al., 2020), and (iii) direct target exons of NOVA and PTBP determined using an integrative modeling approach as reported (Weyn-Vanhentenryck et al., 2018).

### Statistical analysis

For comparisons between basal exon inclusion, or activity-dependent exon inclusion level difference, Pearson correlation coefficients were calculated. Enrichment for targets of particular splicing factors were tested using Fisher’s exact test. Gene ontology enrichment analyses were performed using topGO’s default *weight01* algorithm, which integrates GO graph topology to supplement the Fisher’s exact test used for individual GO terms. Significance of changes in exon inclusion in response to activity were calculated by rMATS, which uses a generalized linear modeling approach to incorporate estimates of per-sample uncertainty, and inter-replicate variability, in PSI into estimates of differential alternative splicing.

## Data Availability

The datasets presented in this study can be found in online repositories. The names of the repository/repositories and accession number(s) can be found below: https://www.ebi.ac.uk/ena, E-MTAB-5489, https://www.ebi.ac.uk/ena, E-MTAB-5514.

## Footnotes

1
https://genome.ucsc.edu/cgi-bin/hgLiftOver
