# Supplementary material for: A comparison of basal and activity-dependent exon splicing in cortical-patterned neurons of human and mouse origin
Source: Front Mol Neurosci. 2024 Aug 29;17:1392408. doi: 10.3389/fnmol.2024.1392408 (PMC11390650; doi:10.3389/fnmol.2024.1392408)
Supplement: Supplementary file 2 [file Data_Sheet_1.pdf]

Supplemental Figures

Figure. S1

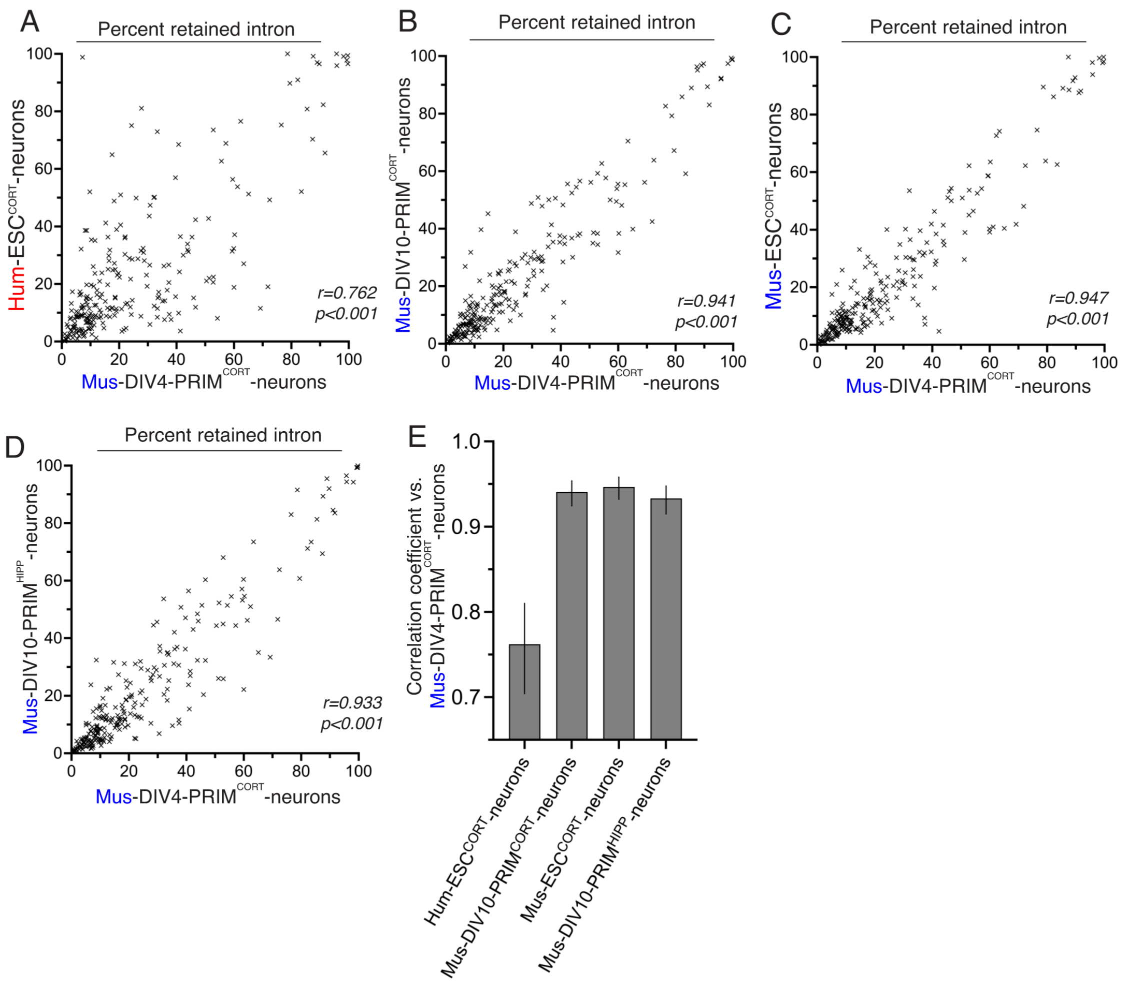

Figure S1, related to Figure 1. A-D) "Percent retained intron" in DIV4 Mus-PRIM^{CORT} neurons plotted against the corresponding percent retained intron in the indicated cell types (mean percent retained intron, n=3 independent biological replicates). All introns plotted have a 1:1 human-mouse ortholog (intron start and intron end could be matched to within ten base-pairs, after translating co-ordinates between the mm10 and hg38 genome assemblies). **E)** Pearson r correlation coefficients for the comparisons made in A-D.

Figure. S2

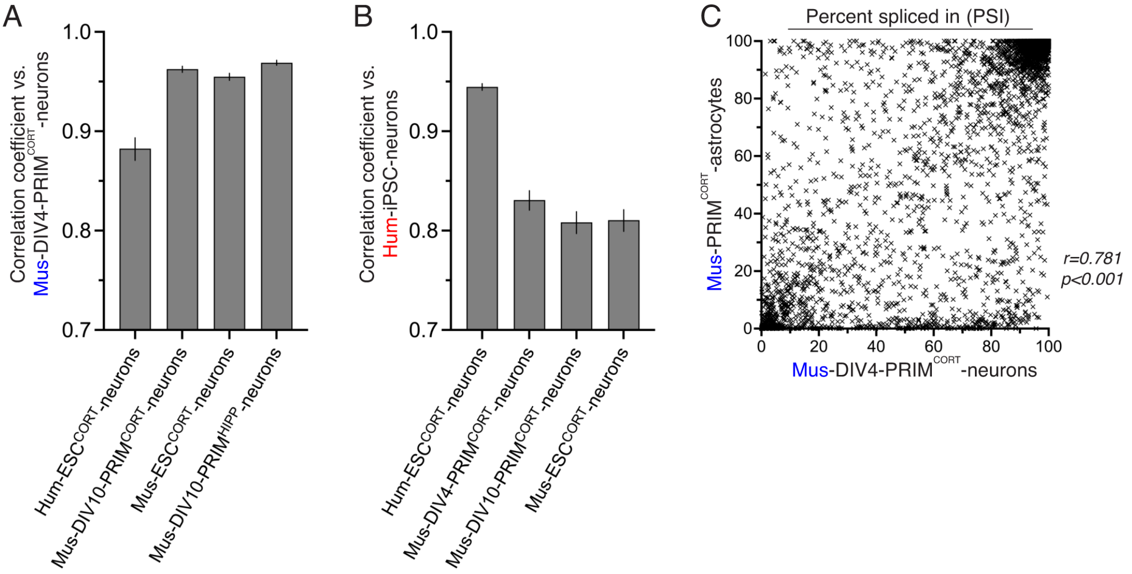

Figure S2, related to Figure 1. A) Pearson r correlation coefficients for the comparisons made in Fig1. A-D, but taking only data from genes expressed at similar levels in Hum-ESC^{CORT} and DIV 4 mouse neurons (within 20% in either direction). Error bars indicate the 95% confidence limits and in all cases $p<0.0001$. **B)** Pearson r correlation coefficient between human iPSC-derived neurons and our Hum-ESC^{CORT} neurons and mouse neuronal samples, for the 1:1 orthologous events shown in Fig. 1A-D. **C)** PSI of exons in DIV4 Mus-PRIM^{CORT} neurons plotted against the corresponding PSI in mouse cortical astrocytes (mean PSI, n=3) for all exons analysed in Fig. 1. For data points relating to this figure see Source_Data.xlsx.

Figure S3

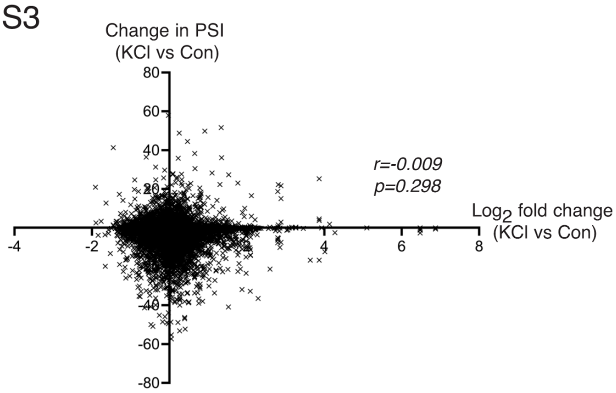

Figure S3, related to Figure 2. In DIV4 Mus-PRIM^{CORT} neurons, the PSI change upon KCl stimulation is plotted against the Log_2 fold-change of the corresponding gene at the mRNA level.
